# Grapevine *DMR6-1* Is a Candidate Gene for Susceptibility to Downy Mildew

**DOI:** 10.3390/biom12020182

**Published:** 2022-01-22

**Authors:** Carlotta Pirrello, Giulia Malacarne, Marco Moretto, Luisa Lenzi, Michele Perazzolli, Tieme Zeilmaker, Guido Van den Ackerveken, Stefania Pilati, Claudio Moser, Lisa Giacomelli

**Affiliations:** 1 Research and Innovation Centre, Fondazione Edmund Mach, Via E. Mach 1, 38098 San Michele all’Adige, Italy; carlotta.pirrello@unipd.it (C.P.); giulia.malacarne@fmach.it (G.M.); marco.moretto@fmach.it (M.M.); lenzi.luisa@gmail.com (L.L.); michele.perazzolli@fmach.it (M.P.); stefania.pilati@fmach.it (S.P.); claudio.moser@fmach.it (C.M.); 2 Department of Agricultural, Food, Environmental and Animal Sciences, University of Udine, Via delle Scienze 206, 33100 Udine, Italy; 3 Center Agriculture Food Environment (C3A), University of Trento, Via E. Mach 1, 38098 San Michele all’Adige, Italy; 4 SciENZA Biotechnologies B.V., Sciencepark 904, 1098 XH Amsterdam, The Netherlands; T.Zeilmaker@enzazaden.nl; 5 Plant-Microbe Interactions, Department of Biology, Utrecht University, Padualaan 8, 3584 CH Utrecht, The Netherlands; g.vandenackerveken@uu.nl

**Keywords:** *DMR6*, *DLO*, downy mildew, *Plasmopara viticola*, *Vitis vinifera*

## Abstract

Grapevine (*Vitis vinifera*) is a valuable crop in Europe for both economical and cultural reasons, but highly susceptible to Downy mildew (DM). The generation of resistant vines is of critical importance for a sustainable viticulture and can be achieved either by introgression of resistance genes in susceptible varieties or by mutation of Susceptibility (S) genes, e.g., by gene editing. This second approach offers several advantages: it maintains the genetic identity of cultivars otherwise disrupted by crossing and generally results in a broad-spectrum and durable resistance, but it is hindered by the poor knowledge about S genes in grapevines. Candidate S genes are *Downy mildew Resistance 6* (*DMR6*) and *DMR6-Like Oxygenases* (*DLO*s), whose mutations confer resistance to DM in Arabidopsis. In this work, we show that grapevine *VviDMR6-1* complements the Arabidopsis *dmr6-1* resistant mutant. We studied the expression of grapevine *VviDMR6* and *VviDLO* genes in different organs and in response to the DM causative agent *Plasmopara viticola*. Through an automated evaluation of causal relationships among genes, we show that *VviDMR6-1*, *VviDMR6-2*, and *VviDLO1* group into different co-regulatory networks, suggesting distinct functions, and that mostly *VviDMR6-1* is connected with pathogenesis-responsive genes. Therefore, *VviDMR6-1* represents a good candidate to produce resistant cultivars with a gene-editing approach.

## 1. Introduction

The control of pathogens in viticulture often relies on the application of massive amounts of pesticides, especially fungicides, which comes at great costs for viticulture and poses a considerable risk for human health and the environment. Cultivated grapevine (*Vitis vinifera* L.) is highly susceptible to diseases such as Downy mildew (DM), caused by the oomycete *Plasmopara viticola*. The production of new resistant grapevine plants relies on breeding programs to introduce dominant Resistance (R) genes in hybrids with desirable traits.

An alternative strategy to obtain resistant plants that has proven successful in several crops is to inactivate the so-called Susceptibility (S) genes, which promote infection and mediate compatible interactions with pathogens [1]. The exploitation of S genes as sources of resistance is limited by the fact that they remain largely unknown in *Vitis*. One popular S gene is *Mildew Locus O* (*MLO*), whose mutations are linked to resistance to powdery mildew in many crops such as barley, tomato, pea, pepper, wheat, and apple [2,3,4,5,6,7,8], indicating that *MLO* provides a general system for resistance across species. In grapevine, downregulation of three *MLO* genes conferred reduced susceptibility to powdery mildew [9].

No S genes are yet known to confer resistance to *P. viticola* in grapevine, although orthologs of the *Arabidopsis thaliana Downy mildew Resistance 6* (*AtDMR6*) are potential candidates. The Arabidopsis *dmr6* mutant was discovered by screening for resistance to *Hyaloperonospora arabidopsidis*, the DM causative agent in this model plant, and later showed resistance also to the oomycete *Phytophthora capsici* and the bacterium *Pseudomonas syringae* [10,11]. *AtDMR6* encodes a 2-oxoglutarate (2OG)-Fe(II) oxygenase, which functions as a negative regulator of immunity. Phylogenetic analysis of the superfamily of 2OG-Fe(II) oxygenases revealed two additional DMR6-Like Oxygenases (AtDLO1 and AtDLO2) closely related to AtDMR6, whose overexpression in the *dmr6* mutant also restored DM susceptibility. AtDMR6 and AtDLO1 are partly redundant in suppressing DM resistance and are involved in Salicylic Acid (SA) inactivation: the *dmr6-dlo1* double mutant is completely resistant to *H. arabidopsidis*, and resistance is accompanied by enhanced expression of defence-related genes, hyper-elevated SA levels, and a corresponding dwarf phenotype. Arabidopsis DMR6 and tomato DMR6-1 were shown to function as SA 5-hydroxylases [12,13], whereas DLO1 was shown to function as an SA 3-hydroxylase [14].

Orthologs of the *DMR6* and *DLO* genes were readily identified in other crops by phylogenetic analysis [11] and complementation of the Arabidopsis *dmr6* mutants. In tomato, inactivation of *DMR6-1*, but not *DMR6-2*, confers broad-spectrum resistance against bacterial and fungal pathogens, by constitutively activating plant immune responses and increasing SA levels [13]. Similarly, in potato, inactivation of *DMR6-1* by CRISPR/Cas9, but not *DMR6-2*, also increased resistance to late blight caused by the oomycete *Phytophthora infestans* [15]. Together, these results suggest different functions of *DMR6* homologs. In addition, recent works proved the role of *DMR6* in susceptibility to Xanthomonas wilt in banana [16] and to *Peronospora belbahrii* in sweet basil [17], suggesting a widely conserved function across the plant kingdom.

In grapevine, the gene family is expanded to five members: two *DMR6* and three *DLO* genes [11,18], but their involvement in DM susceptibility is yet to be demonstrated. Therefore, it is essential to understand which of these genes would be a useful source of resistance when mutated, and at what cost, prior to their exploitation as S genes in lengthy breeding programs or via gene editing. In this work, we provide a preliminary characterisation of the grapevine *DMR6* and *DLO* genes in light of their putative function as S genes.

## 2. Materials and Methods

### 2.1. Multiple-Sequence Alignment and Phylogenetic Analysis

Grapevine DMR6 and DLO protein sequences (gene identifiers in Table S1) were aligned with DMR6 and DLO proteins of other species and of proven function in susceptibility: *Solanum lycopersicum* SlDMR6-1 (Solyc03g080190) and SlDMR6-2 (Solyc06g073080), *Solanum tuberosum* StDMR6 (XP_006347521), *Ocimum basilicum* ObDMR6 (QWT44767.1), *Hordeum vulgare* HvDMR6 (KAE8782493.1), *Zea mays* ZmFNSI (NP_001151167), *Arabidopsis thaliana* AtDLO1 (At4g10500), AtDLO2 (At4g10490), and AtDMR6 (AT5G24530). *Vitis* sequences were retrieved by BLAST against the grapevine reference chromosome assembly (12X.V2) [19]: Vvi16g01336 (*VviDMR6-1*), Vvi13g01119 (*VviDMR6-2*), Vvi15g00871 (*VviDLO1*), Vvi02g00271 (*VviDLO2*), and Vvi02g00270 (*VviDLO3*). A multiple-sequence alignment was generated with MUSCLE with default settings within the Jalview 2 suite [20]. Phylogenetic analysis of the Arabidopsis and *Vitis* sequences, as well as tree drawing were conducted with the PhyML and TreeDyn software, respectively, using the Phylogeny.fr platform (http://www.phylogeny.fr/, last accessed on 18 December 2021) [21] in one-click mode. The PhyML analysis was run with WAG as a substitution model for proteins and SH-like as an aLRT test.

### 2.2. Overexpression of VviDMR6-1 in Arabidopsis thaliana and Complementation

The full-length *VviDMR6-1* coding sequence was cloned in pENTR/D-topo (Invitrogen) and into pK7WG2 [22] by recombination. *Agrobacterium* strain GV3101 carrying the pK7WG2-VviDMR6-1 construct was used to transform *A. thaliana* Col0 plants by floral dipping. T1-positive plants were selected on 1/2 MS plates [23] containing 50 mg/L kanamycin. Four lines overexpressing *VviDMR6-1* (Col0) and the wild-type were planted in soil. Four- to five-week-old T2 plants were spray inoculated with the *H. arabidopsidis* Waco isolate and assessed at 6 d post-inoculation (dpi) in two replicate experiments: eight to ten wild-type plants (Col0) were compared with four 35S::VviDMR6-1 lines (Lines 1 to 4). DM development was expressed as the number of spores collected per plant. The pK7WG2-VviDMR6-1 construct was transformed into *Agrobacterium* strain C58C1, and the presence of the transgene was confirmed by PCR. Arabidopsis *dmr6-1* mutant plants were transformed using the floral dip method. Three T1 lines overexpressing the grapevine transgene in the *dmr6-1* background were selected on plates containing kanamycin, and healthy growing plants were transplanted in soil. Four- to five-week-old T1 plants were spray inoculated with *H. arabidopsidis* isolate Cala 2 and scored at 7 dpi. At least three adult leaves per plant were used to determine the amount of spores per fresh weight.

### 2.3. cDNA Synthesis and qRT-PCR Analysis

RNA was prepared using the Spectrum Plant Total RNA Kit (Merck KGaA, Darmstadt, Germany) according to the manufacturer’s instructions. Total RNA was treated with DNAse I (Thermo Fisher Scientific, Waltham, MA, USA) and then quantified with an ND-8000 spectrophotometer (Thermo-Fisher, Waltham, MA, USA). RNA integrity was checked using the Agilent 4200 TapeStation system (Agilent Technologies, Santa Clara, CA, USA). cDNA was synthesised using either the Superscript VILO™ cDNA Synthesis Kit or Superscript III (Thermo Fisher Scientific, Waltham, MA, USA), according to the manufacturer’s instructions. qRT-PCR analyses were carried out using the KAPA SYBR FAST qPCR Kit (Merck KGaA, Darmstadt, Germany) in a ViiA™ 7 thermocycler (Thermo Fisher Scientific). Plates in the 384-well format were set up according to the sample maximisation strategy [24] with three technical replicates for each reaction. The amplification conditions were: 95 °C for 20 s, followed by 40 cycles of 95 °C for 1 s plus 60 °C for 20 s. Reaction efficiencies were calculated with the LinReg software from non-baseline-corrected data [25]. Reference genes were chosen according to analysis with GeNorm [26]. Relative Quantities (RQs) of expression were divided by a normalisation factor based on the expression of chosen reference genes [27]. Primers used in qRT-PCR analyses are reported in Table S2.

### 2.4. Gene Expression Analysis in Grapevine Organs

Organs of *V. vinifera* cv. Sugraone were collected from three individual plants grown in an experimental field at Fondazione Edmund Mach (San Michele all’Adige, Italy) and were: Young Leaves (YLs) at the developmental stage EL-12 [28], Mature Leaves (MLs; EL-23), Tendrils (TDs; EL-23), and Inflorescences (INF; EL-23). These were split in rachis (RC), ovary (OV) and anthers (AN). Roots (RTs) were collected from cuttings grown in a greenhouse. Samples were placed in liquid nitrogen and stored at −80 °C until use. Relative expression levels were normalised using *VviGAPDH*, *VviACTIN*, and *VviSAND*.

### 2.5. BTH Treatment

In vitro-grown plants (*V. vinifera* cv. Sugraone) were sprayed with 0, 100, and 200 mg/L of benzothiadiazole (BTH, Bion WG Syngenta, 50% active component). Four individual whole plants (biological replicates) were harvested 24 h post-treatment (hpi) and frozen in liquid nitrogen. cDNA synthesis and qRT-PCR were performed as described above. Relative expression levels were normalised using *VviACTIN* and *VviSAND*.

### 2.6. Analysis in Senescent Leaves

Potted plants (*V. vinifera* cv. Brachetto) were grown in greenhouse conditions. Mature leaves and senescing leaves (yellowing) were collected and frozen in liquid nitrogen. cDNA synthesis and qRT-PCR were performed as described above. Relative expression levels were normalised using *VviACTIN*, *VviSAND*, and *VviGADPH*. Two experiments were performed with similar results.

### 2.7. Plasmopara viticola Inoculation Assays and Gene Expression Analysis

*V. vinifera* cv. Sugraone plants were regenerated from callus and propagated in vitro in controlled conditions: 16 h light/8 h dark photoperiod, 23 °C, and 60% Relative Humidity (RH), and then acclimatised in 2 L pots with rooting soil with a low percentage of pumice. Plants were grown from 3–9 months in a greenhouse and then transferred to a controlled growth chamber prior to the artificial inoculation assay. *P. viticola* ((Berk. et Curt.) Berl. et de Toni) was collected in a local vineyard and propagated on grapevine plants. There were three independent inoculation experiments performed, with 10, 20, and 15 plants, respectively. Plants were of similar size and bearing 10–20 leaves. In each experiment, half of the plants were sprayed with a sporangia suspension on the abaxial side of leaves and the other half with distilled water (control). Each plant was then covered with a plastic bag and kept in the dark for 24 h in controlled conditions. Plants were then uncovered and maintained at 16 h light/8 h dark photoperiod, 23 °C, and 60% RH for 6 days until symptoms assessment. To induce sporulation for phenotypic analysis, each plant was covered again with a plastic bag at 7 dpi. *V. riparia* plants were used as negative controls for the infection. Leaves between the fourth and the sixth node were sampled at 0 hpi, 24 hpi, and 96 hpi, frozen in liquid nitrogen, and stored at −80 °C until use. Relative expression levels were normalised using *VviACTIN* and *VviATP16*.

### 2.8. Local Expression of DMR6 and DLO Genes

Leaves of in vitro-grown plants (*V. vinifera* cv. Pinot noir) were treated with water (non-inoculated) or with *P. viticola* (inoculated) under sterile conditions and fixed in 100% acetone at 24 hpi. Guard cells and surrounding cells were dissected using an LMD7000 (Leica Microsystems, Wetzlar, Germany) with the settings described in [29]. Gene expression analysis was carried out on microdissected sectors (guard cells and surrounding cells) and whole fixed leaves (not dissected) of non-inoculated and inoculated (local-inoculated) samples or non-inoculated areas of inoculated leaves (distal-inoculated). Data from two experiments were pooled, as no significant differences between the two experiments were revealed by a factorial ANOVA (*p* > 0.05). Relative expression levels were calculated using *VviACTIN* as the constitutive gene, and the data were calibrated on whole fixed leaves of non-inoculated samples.

### 2.9. Statistical Analyses

In the qRT-PCR analyses, statistically significant differences in gene expression were evaluated in R-studio [30] by ANOVA Tukey-HSD and Fisher’s tests or paired Student’ *t*-tests and Kruskal–Wallis tests where indicated.

### 2.10. In Silico Analysis and Investigation Tools

Expression data of grapevine *DMR6* and *DLO* genes in tissues at different developmental stages were retrieved from the Grape eFP Browser [31] as RPKM values, while expression data relative to experiments with pathogens were retrieved from public gene expression datasets collected in the VESPUCCI compendium [32], normalised as log ratio values, and further analysed with Python within the Google Colab platform. Specifically, experiments relative to the interaction with eight fungal pathogens were selected: *P. viticola* (DM), *Erysiphe necator* (powdery mildew), *Botrytis cinerea* (grey mould), *Neofusicoccum parvum* (trunk disease), *Phaeomoniella chlamydospora* (anthracnose), and *Coniothyrium diplodiella* (white rot), plus the bacterium *Xylella fastidiosa*, the causative agent of Pierce disease, and *Candidatus Phytoplasma vitis*, a Bacteroidetes causing flavescence dorée. Heat maps were produced in Python using the Pandas [33] and seaborn [34] packages. The association network for the *DMR6* and *DLO* genes was obtained using the OneGenE method [35]—which finds causal relationships among genes—based on the transcriptomic data collected in the VESPUCCI compendium [32]. The expansion lists of the five genes of interest were retrieved from Vitis OneGenE (vitis.onegenexp.eu, last accessed on 18 December 2021) and aggregated using the “Expand Network” tool by setting the relative frequency threshold to 0.5. The grapevine *DMR6* and *DLO* expansion network file was imported locally into Cytoscape 3.8.2 [36] for a customised visualisation. For the characterised genes, the annotation was retrieved from the Grapevine Reference Gene Catalogue. For genes already characterised in other species, the annotation was based on homology and was either assigned automatically in VitisNet [37] or retrieved from the literature.

## 3. Results

### 3.1. The Grapevine DMR6-DLO Gene Family

**Figure 1:**
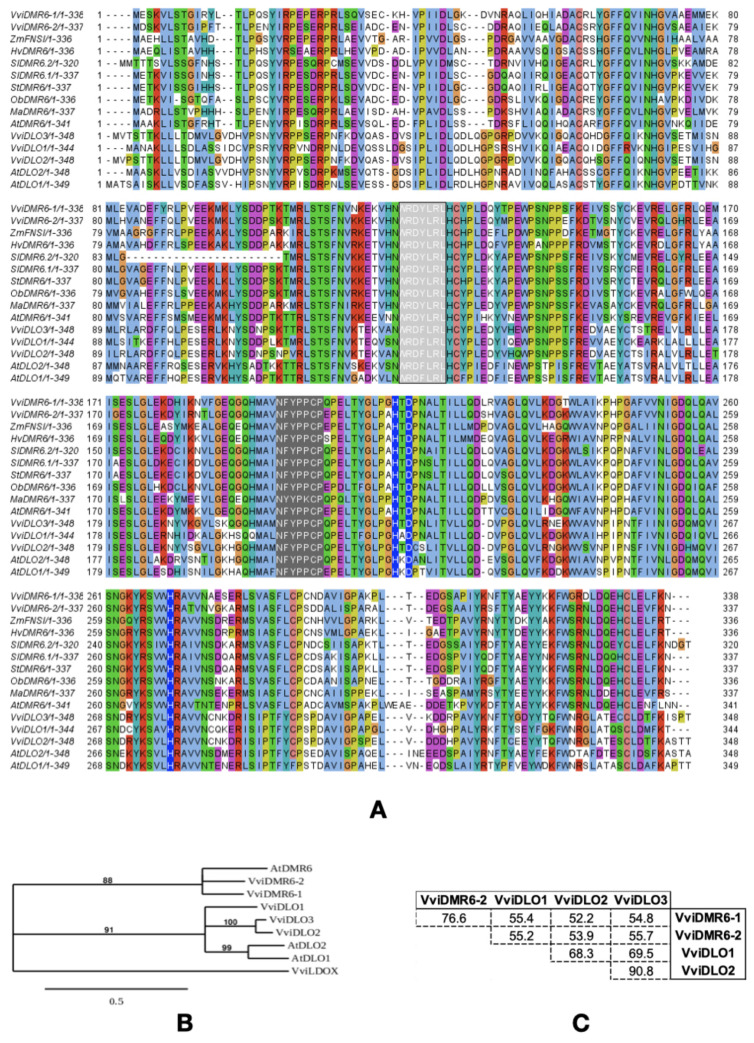
The grapevine DMR6 and DLO protein family. (**A**) Multiple alignment of grapevine DMR6 and DLO proteins with *Zea mays* ZmFNSI (NP_001151167) [40], *Hordeum vulgare* HvDMR6 (KAE8782493.1) [41], *Solanum lycopersicon* SlDMR6-1 (Solyc03g080190) and SlDMR6-2 (Solyc06g073080) [13], *Solanum tuberosum* StDMR6 (XP_006347521) [15], *Ocimum basilicum* ObDMR6 (QWT44767.1) [17], *Musa acuminata* MusaDMR6 (XP_009389864.1) [16], *Arabidopsis thaliana* AtDLO1 (At4g10500), AtDLO2 (At4g10490), and AtDMR6 (AT5G24530) [11]. The alignment is coloured with the default ClustalX colour scheme according to the amino acid chemical properties. The ordering of the sequences is based on pairwise similarity. Motifs that are important for the catalytic function are highlighted: the HDH motif (blue background), the NYYPPCP motif (dark grey), and the WRDY/FLRL motif (light grey)—specific to the DMR6 and DLO proteins. (**B**) Phylogenetic tree of the *V. vinifera* and Arabidopsis DMR6 and DLO sequences. The grapevine leucoanthocyanidin dioxygenase (VviLDOX, Vitvi02g00435) is used as an outgroup. Bootstrap values above 50 are shown. Branches’ length expresses the relative number of amino acid substitutions. (**C**) Amino acid identity (%) within the grapevine DMR6 and DLO sequences.

A phylogenetic analysis of (2OG)-Fe(II) oxygenases in flowering plants reported the existence of a specific clade grouping the DMR6-DLO sequences [11]. Figure 1A shows the alignment of grapevine DMR and DLO proteins with those of Arabidopsis and other species whose role in susceptibility has been confirmed.

Common motifs shared with (2OG)-Fe(II) oxygenases and specific residues in the sequences of the DMR6-DLO clade are highlighted: the HDH motif is a general hallmark of the (2OG)-Fe(II) oxygenases, and it binds the catalytic iron (FeII), while the NYYPPCP motif specifically interacts with the 2-oxoglutarate substrate. The WRDY/FLRL motif—specific to the DMR6 and DLO-type (2OG)-Fe(II) oxygenases—was proposed to be involved in binding SA [38]. Overall, Figure 1A shows a high degree of amino acid conservation. The grapevine proteins form two separate groups with the corresponding *Arabidopsis* orthologs in the phylogenetic tree (Figure 1B). VviDMR6-1 and VviDMR6-2 sequences cluster with AtDMR6 and are separated from the DLO subgroup of grapevine and *Arabidopsis*. From the tree, it is not possible to propose a one-to-one correspondence between the *Vitis* and the *Arabidopsis* DLOs with confidence. The analysis of the pairwise amino acid sequence identity within the five grapevine proteins (Figure 1C) confirmed a high degree of conservation between VviDMR6-1 and VviDMR6-2 (76.6% identity) and between VviDLO2 and VviDLO3 (90.8% identity). VviDLO1 showed a similar degree of identity to the two grapevine DMR6 proteins (about 55% identity) and to the other two DLO proteins (68–69% identity), but is more distant from them than they are to each other. The genes encoding VviDLO2 and VviDLO3 are close to each other in the grapevine genome (about 7 kbp apart), suggesting they originated from a tandem gene duplication.

We propose the nomenclature of the *Vitis* DMR6 and DLO proteins according to the grapevine nomenclature system [39] and based on the third version of the genome annotation [19] as: VviDMR6-1 (Vitvi16g01336), VviDMR6-2 (Vitvi13g01119), VviDLO1 (Vitvi15g00871), VviDLO2 (Vitvi02g00271), and VviDLO3 (Vitvi02g00270), as listed in Table S1.

### 3.2. VviDMR6-1 Is an Ortholog of the A. thaliana DMR6 Gene

**Figure 2:**
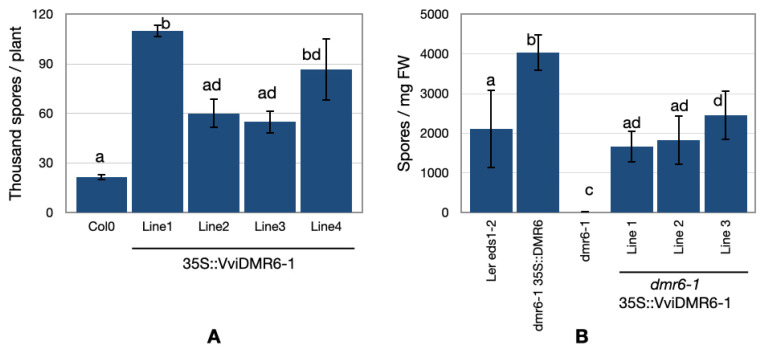
*VviDMR6-1* is an ortholog of the Arabidopsis *DMR6* gene. (**A**) Increased DM-susceptibility of Arabidopsis lines overexpressing *VviDMR6-1*: blue bars represent the spore count (thousand spores) of *H. arabidopsidis* at 6 dpi in four T2 overexpressing lines (Lines 1 to 4). Eight to ten plants per genotype were inoculated and assessed at 6 dpi in two replicate experiments. Error bars represent standard deviations (n = 2). Statistically significant differences are indicated with different letters, according to the Tukey HSD test (*p* < 0.05). (**B**) Complementation of the *dmr6-1* mutant: blue bars represent the spore counts of *H. arabidopsidis* per milligram of leaf (fresh weight). The data include three independent T1 plants (Lines 1 to 3) that show complementation of the *dmr6-1*-resistant phenotype as they become highly susceptible as compared to the parental line (Ler eds1-2, [42]) and complementation line (dmr6-1 35S:DMR6). Error bars represent standard deviations (n = 10). Statistically significant differences are indicated with different letters, according to the Tukey HSD test (*p* < 0.05).

By overexpressing *VviDMR6-1* in *A. thaliana* (Col0), four transgenic lines were obtained, among which Line 1 was the one with the highest expression of the transgene. When inoculated with the oomycete *H. arabidopsidis*, all overexpressing lines showed increased susceptibility to DM as compared to the wild-type (Figure 2A).

Moreover, overexpression of *VviDMR6-1* in the *Arabidopsis dmr6-1* mutant (dmr6-1 35S::VviDMR6-1) reverted the susceptible phenotype (Figure 2B) to a level comparable to that of its parental line (*Landsberg erecta* eds1-2) and the complementation line (dmr6-1 35S:DMR6), indicating that *VviDMR6-1* may be involved in DM susceptibility in grapevine.

### 3.3. Expression of DMR6-DLO Gene Family in Grapevine Tissues

**Figure 3:**
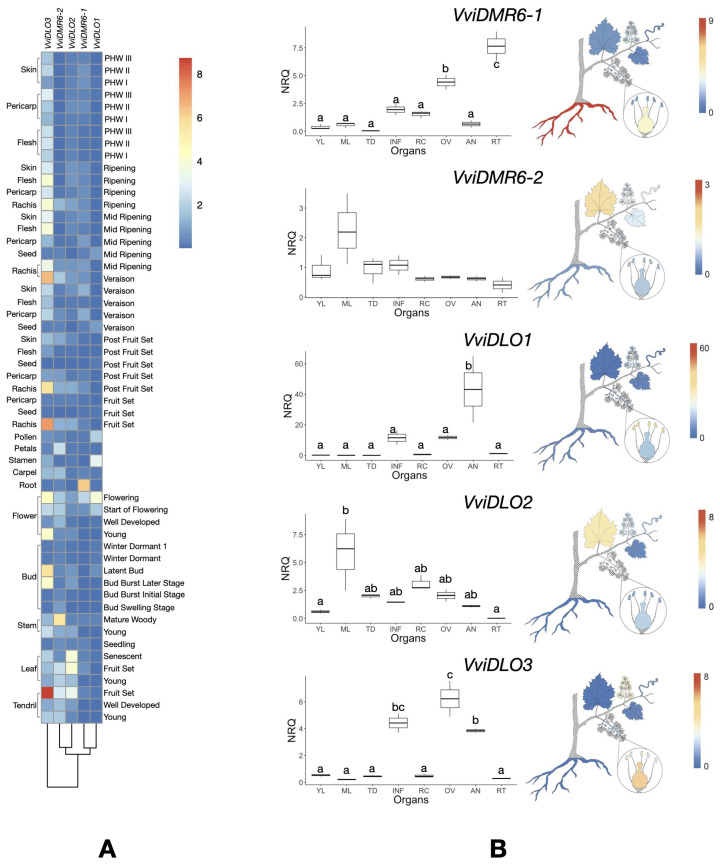
Expression of the *DMR6* and *DLO* genes in grapevine organs. (**A**) Heat map of grapevine *DMR6* and *DLO* expression in organs at different developmental stages (PHW: Post-Harvest Withering). Absolute RPKM values were downloaded from the Grape eFP Browser [31] and normalised across conditions. (**B**) Boxplots indicate Normalised Relative Quantities (NRQs) of grapevine *DMR6* and *DLO* mRNAs analysed by qRT-PCR in the following organs: Young Leaves (YLs), Mature Leaves (MLs), Tendrils (TDs), Inflorescence (INF), Rachis (RCs), Ovaries (OVs), Anthers (ANs), and Roots (RTs). Statistically significant differences are indicated with different letters, according to the Tukey HSD test (*p* < 0.05). The same results are visualised in the drawings on the right side by the colour scale.

To study the expression of grapevine *DMR6* and *DLO* genes, we performed an in silico analysis of publicly available grapevine transcriptomic data obtained in *V. vinifera* cv. Corvina (Figure 3A). In this dataset, *VviDMR6-1* is abundantly expressed in roots, *VviDMR6-2* in stems, *VviDLO1* in stamen and pollen, and *VviDLO2* in tendrils and leaves, while *VviDLO3* shows a high expression in tendrils of plants at the fruit-set stage and in the rachis at almost all phenological stages at fruit-set and later stages. *VviDLO3* expression in rachis at anthesis and tendrils of a young plant is much lower than that of the inflorescence. Grapevine *DMR6* and *DLO* genes are poorly expressed in fruits, with the exception of *VviDLO3*, which is expressed in fruit tissues during ripening. To better investigate their expression in organs other than fruit, their basal expression was further studied in vegetative organs and flowers of *V. vinifera* cv. Sugraone (Figure 3B) by qRT-PCR analysis. In accordance with the in silico analysis, *VviDMR6-1* showed the highest expression in roots and in the ovary at anthesis and a poor expression in leaves and tendrils. *VviDMR6-2* and *VviDLO2* were both highly expressed in mature leaves. No significant differences in *VviDMR6-2* expression were observed among all the other tissues. *VviDLO2* was highly expressed also in the rachis and the ovary. *VviDLO1* and *VviDLO3* transcripts were almost undetectable or very poorly represented in the leaves, tendrils, roots, and rachis, while they were highly expressed in the flower: *VviDLO1* was especially expressed in anthers, whereas *VviDLO3* expression seemed restricted to anthers and ovaries.

### 3.4. Induction of DMR6-DLO Genes by BTH and Senescence

**Figure 4:**
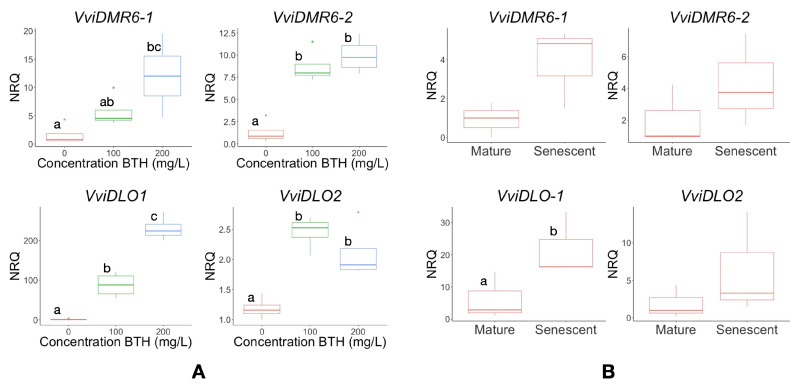
Expression of grapevine *DMR6* and *DLO* genes in BTH-treated leaves and during senescence. (**A**) Boxplots of Normalised Relative Quantities (NRQs) of *VviDMR6-1*, *VviDMR6-2*, *VviDLO1*, and *VviDLO2* transcripts detected in four in vitro plants treated with 0 mg/L, 100 mg/L, and 200 mg/L of BTH and harvested after 24 h. Different letters indicate significant differences according to an ANOVA Tukey test (*p* < 0.05). (**B**) Boxplots show the results of one of two experiments with similar results: NRQ of *VviDMR6-1*, *VviDMR6-2*, *VviDLO1*, and *VviDLO2* transcripts in mature and senescent leaves harvested from three individual plants. Different letters indicate significant differences according to a paired Student’s test (*p* < 0.05). Letters were omitted in the case of non-significant differences.

To investigate the response of *DMR6* and *DLO* genes to SA, we analysed their expression in plants treated with the SA analogue BTH (Figure 4A). In vitro plants were chosen for this experiment to ensure the absence of pathogens, which may alter gene expression. The *VviDLO3* transcript was often undetected in leaves and was therefore excluded from this and further qRT-PCR analyses. The *DMR6* and *DLO* genes were strongly induced in plants sprayed with increasing concentrations of BTH (Figure 4A) at 24 h after the treatment.

In addition, we investigated the response of these genes during senescence. Mature (fully expanded) leaves were compared with senescent (yellowing) leaves of potted plants. All four genes tested were induced in senescent leaves (Figure 4B), although only the induction of *VviDLO1* was significant.

### 3.5. Expression of DMR6-DLO Genes in Different Leaf Sectors under Pathogen Pressure

The expression of *VviDMR6-1*, *VviDMR6-2*, *VviDLO1*, and *VviDLO2* was evaluated upon *P. viticola* infection in the leaves of potted plants (*V. vinifera* cv. Sugraone) grown in greenhouse conditions. *VviDMR6-1*, *VviDMR6-2*, and *VviDLO1* showed a trend of slight induction between control and treated samples both at 24 hpi and 96 hpi, although statistically not significant. High variability was observed among different plants and experiments (Figure S1).

**Figure 5:**
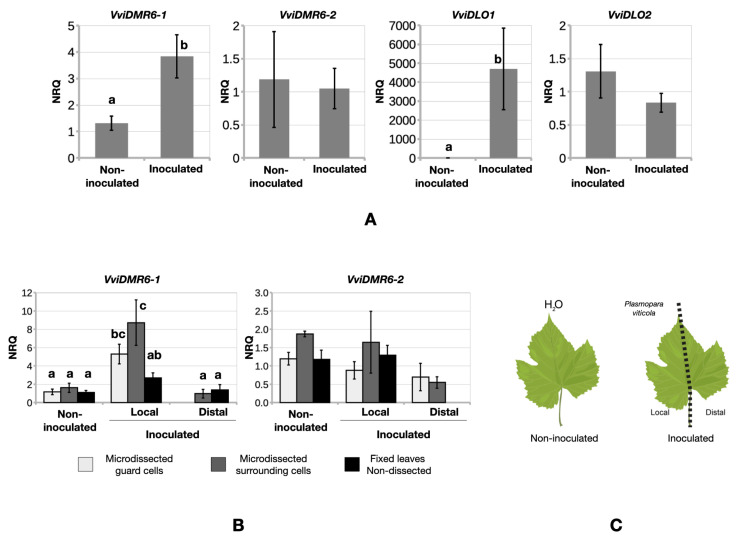
Expression analysis in leaf sectors under *P. viticola* pressure. (**A**) NRQs of *VviDMR6-1*, *VviDMR6-2*, *VviDLO1*, and *VviDLO2* transcripts in fresh leaves either inoculated with *P. viticola* at 24 hpi or non-inoculated. (**B**) NRQs of *VviDMR6-1* and *VviDMR6-2* in leaves treated with water (non-inoculated) or with *P. viticola* (inoculated). Gene expression analyses were carried out on microdissected sectors (0.7–0.8 mm^2^): guard cells, surrounding cells, and fixed leaves (non-dissected). (**C**) Microdissected samples were harvested from non-inoculated samples, inoculated (local-inoculated), or non-inoculated areas of inoculated leaves (distal-inoculated). Mean NRQ levels and standard error values from eight replicates pooled from two experiments are presented for each sample. Different letters indicate significant differences according to Fisher’s test (*p* < 0.05). Letters are omitted in the cases of non-significant differences.

An additional qRT-PCR expression analysis was performed on in vitro-grown plants (cv. P. noir) challenged with *P. viticola* and in controlled conditions, in an attempt to reduce the variability between replicates. At 24 hpi, only *VviDMR6-1* and *VviDLO1* were induced in leaves inoculated with the pathogen (Figure 5A). The expression of *VviDMR6-1*, *VviDMR6-2*, *VviDLO1*, and *VviDLO2* was also evaluated in laser-microdissected leaf sectors. *VviDMR6-1* was induced by *P. viticola* in microdissected guard cells and surrounding cells of locally inoculated samples (Figure 5B,C), while *VviDMR6-2* was not modulated by *P. viticola* (Figure 5A,B). *VviDLO1* and *VviDLO2* were not consistently amplified in microdissected samples, possibly due to low expression levels.

### 3.6. Gene Expression Meta-Analysis of the Grapevine DMR6 and DLO Genes in Response to Pathogens

**Figure 6:**
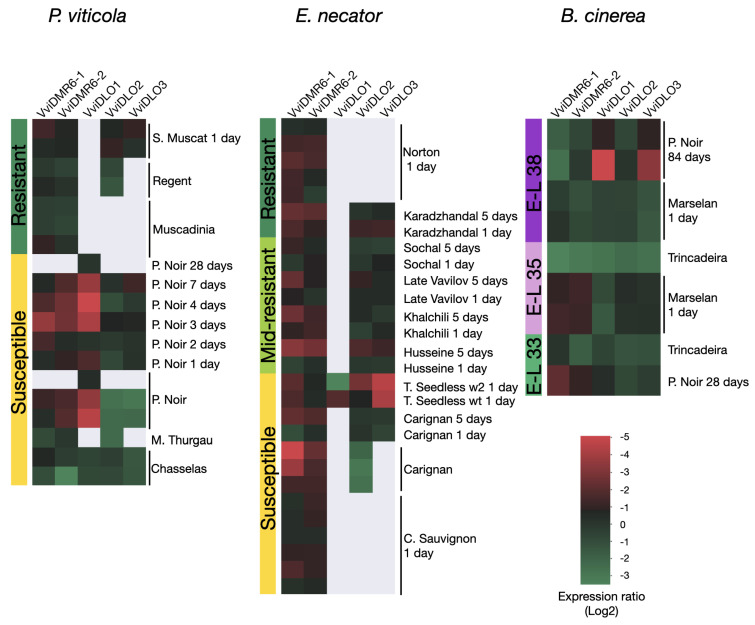
Gene expression meta-analysis of grapevine *DMR6* and *DLO* genes in different pathosystems. The heat plots display the distribution of the expression values of grapevine *DMR6* and *DLO* genes across experiments investigating the response to *P. viticola*, *E. necator*, and *B. cinerea* and collected in VESPUCCI. The expression values are calculated as ratios on a log2 scale between a sample and its reference: either a healthy and an infected sample or between the indicated time point and the time point of the inoculation with the pathogen. The expression values were grouped according to cultivar resistance in the first two cases and on the basis of berry developmental stages in the third case. The NCBI accession numbers of the considered experiments are reported in Table S3.

On the basis of the obtained evidence of a significant induction of *VviDMR6-1* and *VviDLO1* in leaves infected with *P. viticola*, we further investigated the involvement of grapevine *DMR6* and *DLO* genes in the response to other pathogens by mining the VESPUCCI compendium [32,43]: an *in-house*-developed *Vitis* database, which allows the comparison of expression data from different datasets. Specifically, we retrieved expression data relative to experiments with ten pathogens (eight fungi, one bacterium, and one phytoplasma) responsible for the most common grapevine diseases. The most interesting results are summarised in Figure 6 and involve experiments with *P. viticola*, *E. necator*, and *B. cinerea*, whereas the complete output of the analysis is reported in Table S3. In the grapevine–*P. viticola* pathosystem, we observed that *VviDMR6-1*, *VviDMR6-2*, and *VviDLO1* are moderately upregulated upon infection with *P. viticola* and that the modulation of *VviDMR6-1* and *VviDMR6-2* is of higher amplitude in susceptible cultivars as compared to resistant cultivars (Figure 6). In experiments with the ascomycete *E. necator*, *VviDMR6-1* and *VviDMR6-2* are slightly induced in all the cultivars, with no evident difference in response between susceptible and resistant cultivars. In the case of experiments with *B. cinerea*, the expression data were grouped according to berry developmental stages, since ripening promotes the spreading of this ascomycete. In general, these five genes do not seem to be modulated by *Botrytis* infection, especially from veraison onward, with the exception of *VviDMR6-1*, whose expression is the highest at the green berry stage.

### 3.7. Gene Network Analysis

**Figure 7:**
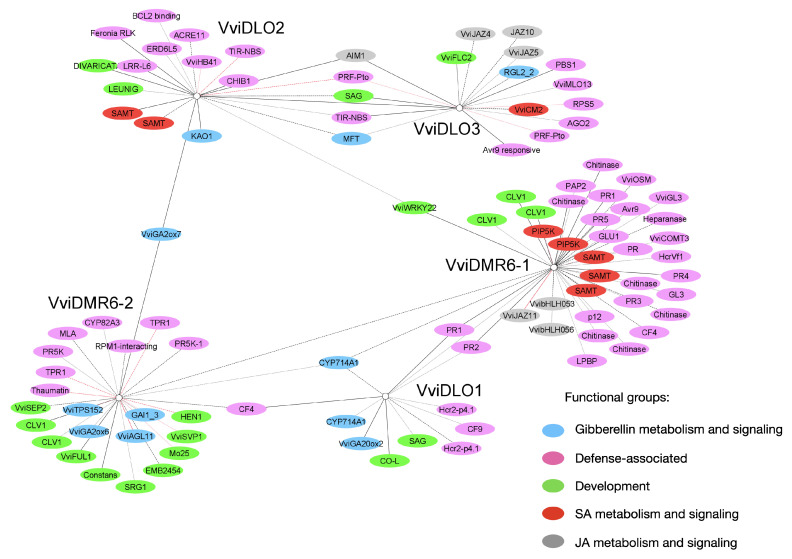
Gene network analysis of grapevine *DMR6* and *DLO* genes. Genes are represented as nodes, and their positive or negative correlations are represented by black and red edges, respectively. For better visualisation, only genes of the most interesting and populated functional categories are shown. Grouping into functional categories is based on manual curation of the gene list and is indicated by node colouring: defence (pink), SA metabolism and signalling (red), GA metabolism and signalling (cyan), jasmonic acid (JA) metabolism and signalling (grey), and development—including senescence (green). Line types (solid, dashed, or dotted) represent the edge weight class, which is based on the relative frequency (decreasing from 1 to 0.5).

To gain insight into the function of grapevine *DMR6* and *DLO* genes, we reconstructed their gene association network by identifying their directly interacting genes. For the scope, we used the OneGenE method [35], which finds causal relationships among genes based on transcriptomic data of the *Vitis* compendium VESPUCCI [32,43]. The obtained network consists of 764 genes (listed in Table S4), including 18 gene clusters as defined by VESPUCCI and 133 unknown genes. The functional annotation of the remaining 613 genes was improved by manual curation. The visualisation of the *DMR6*–*DLO* gene network shows two clusters of genes: a three-centred, sparsely connected subnetwork composed by the *VviDMR6-1*, *VviDMR6-2*, and *VviDLO1* interacting genes and a distinct subnetwork formed by *VviDLO2* and *VviDLO3*, sharing 41 genes and representing 21% and 24% of their respective gene sets (Figure S2). A simplified version of the network is shown in Figure 7. It includes the most populated functional categories (grouped by colour) and genes with solid functional annotation (of proven function in grapevine or based on homology with known genes of other species). We identified 53 defence-associated genes, 28 of which are in the *VviDMR6-1* subnetwork (Figure 7), 11 genes involved in gibberellin (GA) metabolism and signalling, and 15 genes involved in jasmonic and salicylic acid metabolism and signalling (7 and 8, respectively). In addition, 20 genes are annotated as involved in development and are especially found in the *VviDMR6-2* subnetwork (Table S4). These genes and their functional annotation are listed in Table 1.

## 4. Discussion

In Arabidopsis, *AtDMR6* and *AtDLO1* both function as SA hydroxylases in controlling SA homoeostasis and abiotic and biotic stresses. Mutations in *AtDMR6* produce a much stronger resistance than mutations in *AtDLO1* (or *AtDLO2*), suggesting a primary role of *AtDMR6* in defence [10,11]. With the expansion of the family in other crops (such as grapevine) (Figure 1), functional differences are found even within the *DMR6* genes. In Solanaceae, where the DLO1 clade is missing, only *DMR6-1*, but not *DMR6-2*, was shown to function as an S gene [13,15].

By complementing the Arabidopsis DM-resistant mutant *dmr6-1*, we showed that *VviDMR6-1* is the functional ortholog of *AtDMR6* and may be exploited to generate DM-resistant plants (Figure 2). In Arabidopsis, the different effectiveness of *AtDMR6* and *AtDLO1* in their level of DM-resistance is partially explained by their different localisation upon pathogen invasion: *AtDMR6* expression is induced locally in cells surrounding the DM-invaded tissue, whereas *AtDLO1* is induced along the veins [11]. Using a microdissection technique, we showed that also in grapevine, *VviDMR6-1* is induced by the pathogen, especially at the site of the infection (Figure 5), suggesting a role of the VviDMR6-1 protein at the early stages of pathogen invasion.

We investigated the induction of the grapevine *DMR* and *DLO* genes in different pathosystems both mining available public data and performing ad hoc infection experiments with plants in pots and in vitro. These analyses highlighted an induction, though moderate, of *VviDMR6-1* upon infection with the oomycete *P. viticola* (Figure 5A). No differential expression was observed in the DM-resistant *Vitis* genotypes, such as *V. vinifera* cv. Regent and the *V. rotundifolia* cv. Muscadinia (Figure 6). A difference in modulation between susceptible and resistant genotypes was also reported in banana, where *MusaDMR6* is induced by Xanthomonas wilt in the susceptible *Sukali Ndiizi* and not induced in the resistant variety *Musa balbisiana* [16].

Notably, a certain modulation of both grapevine *DMR6* genes was observed in infection experiments with the ascomycete *E. necator*, the causative agent of powdery mildew (Figure 6), suggesting a role in the broad response to more than one pathogen, as it was observed in tomato and Arabidopsis [11,13]. The genes under study also respond to BTH treatment, an SA analogue, known to play an important role in signalling during plant biotic stresses (Figure 4) [78].

Interestingly, also the network analysis suggested a primary role of *VviDMR6-1* in susceptibility, given that 17% of the genes in its network are annotated as defence associated (Table 1). *VviDMR6-1* shares a regulatory network with a number of genes encoding Pathogenesis-Related (PR) proteins. In addition, the *VviDMR6-1* subnetwork contains genes encoding germin-like proteins—e.g., VviGL3, known to be induced by *E. necator* and expressed at the site of infection [70], two beta glucanases associated with resistance to *P. viticola* [53], and the elicitor response protein Avr9 [45]. Moreover, several genes of this network are known to be associated with defence in other species: the *Homologous to Cladosporium fulvum resistance* (HcrVf1), associated with scab resistance in apple [54]; the blight-associated protein P12 known to confer blight resistance in citrus [58]; Phosphatidic Acid Phosphatase (PAP2) known to confer resistance to *Ralstonia solanacearum* in tobacco [59]; heparanases that are induced by powdery mildew in barley [55]; the *CF4* resistance gene of tomato [47] shared also with the subnetworks of *VviDMR6-2* and *VviDLO1*. It is worth mentioning that an RNA-Seq analysis of *Sldmr6-1* lines in tomato treated with *Xanthomonas gardneri* reported the slight, but significant modulation even in mock conditions of about a thousand genes in the mutant versus the wild-type, and the most remarkable changes occurred in genes related to plant immunity such as receptors, *PR* genes, *WRKY* transcription factors, and genes related to SA response [13].

However, *DMR6* unlikely evolved to render a plant more susceptible to a pathogen. Assuming a conserved primary function as SA hydroxylase in grapevine, it remains to be understood what the role of *VviDMR6-1* is in roots, where this gene is mostly expressed (Figure 3). A high expression in roots was also observed for the tomato *SlDMR6-1* gene [13].

A few defence-associated genes are also present in the network of *VviDMR6-2*: besides the already mentioned *CF4*, there are two genes encoding TOPLESS-related proteins. In Arabidopsis, TOPLESS interacts with the (TIR)-NB-LRR R protein SNC1 to repress negative regulators of immunity [68]. In addition, there are a gene encoding RPM1 interacting protein 4, which functions in plant immunity in Arabidopsis [67], the MLA resistance protein, which is a PM resistance protein in barley [57], CYP82A3, a soy bean cytochrome P450 involved in resistance to pathogens and response to stresses [51], and two PR5K proteins, which regulate plant immunity towards *Sclerotinia sclerotiorum* in *Brassica napus* [65].

The most represented functional category in the network of *VviDMR6-2* is development (7%), followed by defence associated (5%), suggesting that this gene may also contribute to susceptibility to pathogens. *VviDMR6-2* does not appear to be induced by DM, locally or systemically at 24 hpi (Figure 5B), but is likely induced at later time points (Figure 6).

Additional defence-associated genes are found in the *VviDLO1* network: besides the already mentioned *PR1*, *PR2*, and *CF4*, there are the resistance gene *CF9*, two genes annotated as the *Homologous to Cladosporium fulvum Resistance* genes (*HCR2*), and two WRKY transcription factors (WRKY08 and 25) known to be induced by pathogens [80], although their association with defence is not clear. Moreover, MYB108 is closely related to the ABA-dependent *Botrytis Susceptible 1* gene, which is a negative regulator of cell death triggered by wounding or pathogens [81,82].

*AtDLO1* was first isolated as a senescence-associated gene and shown to regulate longevity by mediating SA catabolism [14]. In grapevine, the expression of *VviDLO1* seems to be restricted to the flower, whereas in leaves, *VviDLO1* is barely expressed in unchallenged conditions and strongly activated by pathogens. It is connected with defence-associated genes and with two pathogen-induced WRKY transcription factors (Table S4), suggesting a marginal role in susceptibility as well.

Given their high similarity, their partly overlapping networks, and similar trends of expression in response to pathogens, *VviDLO2* and *VviDLO3* may have similar functions in different organs (namely leaves and flowers).

It is worth noting that genes involved in GA metabolism and signalling are shared among subnetworks and are especially connected with *VviDMR6-2*. They encode enzymes involved in the maintenance of the pool of biologically active GAs, and therefore in the biosynthesis of active GAs such as *ent*-kaurene synthase, *ent*-kaurenoic acid oxidase, and GA20ox2 [75], as well as genes encoding GA-deactivating enzymes such as GA2ox6 and GA2ox7 and two GA carboxylases (CYP7141A1). In addition, two genes involved in GA signalling (*VviGAI1-3* and *VviRGL2*) are in the *VviDMR6-2* and *VviDLO3* subnetworks, respectively. A GA-responsive gene is also shared between the *VviDLO2* and *VviDLO3* subnetworks: *Mother of Flowering Locus T* (*MFT*) involved in flower development. SA-mediated alteration of the GA metabolic and signalling pathway was previously shown to occur in response to abiotic stresses in Arabidopsis seeds and during virus infection in potato [83,84].

In conclusion, considering that all five grapevine *DMR6*s and *DLO*s are connected to defence-related genes in their network, it is tempting to suggest that the mutation of more than one gene together with *VviDMR6-1* would provide a stronger resistance phenotype. However, this may come at the expenses of growth and development, considering the connection of all these genes with GA metabolism and signalling. Whether abolishing the sole *VviDMR6-1* function is sufficient to obtain resistance remains to be determined, as well as whether undesirable pleiotropic effects are to be expected. Functional studies of each *DMR6* and *DLO* gene in grapevine may help answering these questions.

## Figures and Tables

**Table 1:** Selection of annotated genes of the grapevine *DMR6* and *DLO* network. The Gene Identifiers (Gene IDs) relative to the V1 prediction and the protein names are provided, together with functional annotation, citations to supporting literature when available, and the subnetwork. DEF: associated with Defence, JA: Jasmonic Acid metabolism and signalling, SA: Salicylic Acid metabolism and signalling, GA: Gibberellin metabolism and signalling, DEV: Development, fDEV: flower Development, SEN: Senescence.

Gene ID	Name	Functional Annotation	Citation	Subnetwork(s)
VIT_11s0016g04840	ACRE11 (Avr9-Cf9 Rapidly Elicited)	DEF	[44]	DLO2
VIT_10s0042g01180	AGO2 (ARGONAUTE 2)	DEF		DLO3
VIT_07s0031g03090	Avr9 (Elicitor Protein)	DEF	[45]	DMR6-1
VIT_18s0001g04150	Avr9 Responsive	DEF		DLO3
VIT_10s0003g04270	BCL2 Binding Anthogene	DEF	[46]	DLO2
VIT_18s0089g00650	CF4	DEF	[47]	DMR6-1
VIT_19s0027g01230	CF4	DEF	[47]	DMR6-2, DLO1
VIT_19s0085g00160	CF9	DEF	[48]	DLO1
VIT_04s0008g00140	CHI28 (Chitinase)	DEF		DMR6-1
VIT_05s0094g00300	Chitinase	DEF		DMR6-1
VIT_05s0094g00320	Chitinase	DEF		DMR6-1
VIT_05s0094g00330	Chitinase	DEF		DMR6-1
VIT_05s0094g00340	Chitinase	DEF	[49]	DMR6-1
VIT_16s0050g02230	Chitinase	DEF		DMR6-1
VIT_15s0046g01600	CHIB1 (Chitinase)	DEF	[50]	DLO2
VIT_18s0001g11470	CYP82A3	DEF	[51]	DMR6-2
VIT_14s0030g00220	ERD6L5 (Sugar Transporter)	DEF		DLO2
VIT_14s0060g02760	GL3 (Germin-Like 3)	DEF	[52]	DMR6-1
VIT_05s0077g01150	GLU1 (Beta-1;3-Glucanase)	DEF	[53]	DMR6-1
VIT_06s0004g00310	Hcr2-p4.1	DEF		DLO1
VIT_06s0004g00330	Hcr2-p4.1	DEF		DLO1
VIT_01s0010g03210	HcrVf1	DEF	[54]	DMR6-1
VIT_11s0118g00420	Heparanase	DEF	[55]	DMR6-1
VIT_16s0100g00270	LPBP (Peptidoglycan Binding)	DEF		DMR6-1
VIT_18s0041g00190	LRR-L6	DEF	[56]	DLO2
VIT_18s0001g11250	MLA	DEF	[57]	DMR6-2
VIT_11s0016g04720	P12 (Blight Associated)	DEF	[58]	DMR6-1
VIT_06s0004g05800	PAP2	DEF	[59]	DMR6-1
VIT_01s0146g00250	PBS1 (avrPphB Susceptible 1)	DEF		DLO3
VIT_19s0014g01180	PR (Pathogenesis Related)	DEF		DMR6-1
VIT_03s0088g00780	PR1	DEF	[60]	DMR6-1
VIT_03s0088g00810	PR1	DEF	[60]	DMR6-1, DLO1
VIT_08s0007g06060	PR2 (Beta-1;3-Glucanase)	DEF	[61,62]	DMR6-1, DLO1
VIT_04s0008g00120	PR3 (Chitinase, CHI27)	DEF	[61,62]	DMR6-1
VIT_14s0081g00030	PR4	DEF	[61]	DMR6-1
VIT_02s0025g04320	PR5 (Thaumatin-Like)	DEF	[63,64]	DMR6-1
VIT_01s0011g03900	PR5K	DEF	[65]	DMR6-2
VIT_17s0000g03320	PR5K-1	DEF	[65]	DMR6-2
VIT_03s0038g01520	PRF-Pto	DEF	[66]	DLO2, DLO3
VIT_15s0046g02810	PRF-Pto	DEF	[66]	DLO3
VIT_00s0231g00040	RPM1-Interacting Protein 4	DEF	[67]	DMR6-2
VIT_09s0002g05070	RPS5	DEF		DLO3
VIT_03s0038g02160	Thaumatin	DEF		DMR6-2
VIT_18s0117g00400	TIR-NBS	DEF		DLO2
VIT_18s0001g06160	TIR-NBS	DEF		DLO2, DLO3
VIT_04s0008g06390	TPR1 (Topless-Related 1)	DEF	[68]	DMR6-2
VIT_04s0008g06400	TPR1 (Topless-Related 1)	DEF	[68]	DMR6-2
VIT_16s0098g00850	VviCOMT3 (Caffeic Acid O-Methyltransferase)	DEF	[69]	DMR6-1
VIT_14s0128g00690	VviGL3 (Germin-Like)	DEF	[52,70]	DMR6-1
VIT_11s0103g00650	VviHB41	DEF		DLO2
VIT_06s0004g03100	VviMLO13	DEF		DLO3
VIT_02s0025g04280	VviOSM (Osmotin-Like)	DEF	[61,64]	DMR6-1
VIT_01s0244g00090	FERONIA RLK (FERONIA Receptor-Like Kinase)	DEF, JA	[71]	DLO2
VIT_04s0008g00310	CLV1 (CLAVATA)	DEV	[72]	DMR6-1
VIT_04s0008g00370	CLV1 (CLAVATA)	DEV	[72]	DMR6-1
VIT_04s0008g00410	CLV1 (CLAVATA)	DEV	[72]	DMR6-1
VIT_04s0008g00390	CLV1 (CLAVATA)	DEV	[72]	DMR6-2
VIT_04s0008g00440	CLV1 (CLAVATA)	DEV	[72]	DMR6-2
VIT_01s0011g03820	EMB2454 (Embryo Defective)	DEV		DMR6-2
VIT_17s0000g01640	EMB2746 (Embryo Defective)	DEV		DLO2
VIT_19s0090g01550	Mo25	DEV		DMR6-2
VIT_12s0059g02500	CONSTANS	f-DEV		DLO1
VIT_07s0104g01360	CONSTANS	f-DEV		DMR6-2
VIT_19s0015g01170	DIVARICATA	f-DEV		DLO2
VIT_10s0003g05070	HEN1 (HUA Enhancer 1)	f-DEV		DMR6-2
VIT_07s0005g06380	LEUNIG	f-DEV		DLO2
VIT_14s0068g01800	VviFLC2 (SEPALLATA 3)	f-DEV		DLO3
VIT_17s0000g04990	VviFUL1 (APETALA 1)	f-DEV		DMR6-2
VIT_17s0000g05000	VviSEP2 (SEPALLATA 1)	f-DEV		DMR6-2
VIT_00s0313g00070	VviSVP1 (Short Vegetative Phase 1)	f-DEV		DMR6-2
VIT_03s0167g00190	CYP714A1 (GA Carboxylase)	GA	[73]	DLO1
VIT_18s0089g00700	CYP714A1 (GA Carboxylase)	GA	[73]	DMR6-1, DMR6-2, DLO1
VIT_01s0011g05260	GAI1_3 (DELLA Protein)	GA		DMR6-2
VIT_06s0004g05050	KAO1 (Ent-Kaurenoic Acid oxidase)	GA	[74]	DLO2
VIT_00s0203g00080	MFT (Mother of Flowering Locus T)	GA		DLO2, DLO3
VIT_07s0005g01500	RGL2_2 (DELLA RGA-Like 2)	GA		DLO3
VIT_18s0041g01880	VviAGL11 (SEEDSTICK)	GA		DMR6-2
VIT_04s0044g01650	VviGA20ox2	GA	[75]	DLO1
VIT_19s0177g00030	VviGA2ox6	GA	[75]	DMR6-2
VIT_10s0116g00410	VviGA2ox7	GA	[75]	DMR6-2, DLO2
VIT_19s0085g00830	VviTPS152 (Ent-Kaurene Synthase)	GA	[74]	DMR6-2
VIT_13s0047g00450	VvibHLH053	JA		DMR6-1
VIT_13s0073g00400	VvibHLH056	JA		DMR6-1
VIT_01s0146g00480	JAZ10	JA		DLO3
VIT_17s0000g02230	VviJAZ11	JA		DMR6-1
VIT_09s0002g00890	VviJAZ4	JA		DLO3
VIT_10s0003g03790	VviJAZ5	JA		DLO3
VIT_11s0016g03690	AIM1	JA, SA	[76]	DLO2, DLO3
VIT_02s0012g00090	PIP5K (Phosphatidylinositol 4-Phosphate 5-Kinase)	SA	[77]	DMR6-1
VIT_08s0007g04700	PIP5K (Phosphatidylinositol 4-Phosphate 5-Kinase)	SA	[77]	DMR6-1
VIT_01s0011g05890	SAMT	SA		DMR6-1
VIT_12s0057g01070	SAMT	SA		DMR6-1
VIT_17s0000g02870	SAMT	SA	[78]	DMR6-1
VIT_04s0023g02260	SAMTBSCMT	SA		DLO2
VIT_18s0001g12900	SAMTBSCMT	SA		DLO2
VIT_04s0008g06570	VviCM2 (Chorismate Mutase)	SA		DLO3
VIT_14s0006g01210	SAG (Senescence-Associated Gene)	SEN		DLO1
VIT_09s0002g00420	SAG (Senescence-Associated Gene)	SEN		DLO2, DLO3
VIT_05s0020g01310	SRG1 (Senescence Related Gene)	SEN	[79]	DMR6-2
VIT_07s0031g01710	VviWRKY22	SEN	[80]	DMR6-1, DLO2
VIT_05s0077g00500	VviMYB108A		[81,82]	DLO1
VIT_04s0008g05760	VviWRKY08		[80]	DLO1
VIT_08s0058g01390	VviWRKY25		[80]	DLO1

## Data Availability

Expression data of grapevine genes in tissues at different developmental stages were retrieved from the Grape eFP Browser at http://bar.utoronto.ca/efp_grape/cgi-bin/efpWeb.cgi (last accessed on 18 December 2021). Grapevine *DMR6* and *DLO* expression data relative to experiments with pathogens were retrieved from public gene expression datasets collected in VESPUCCI, https://vespucci.readthedocs.io/en/latest/ (last accessed on 18 December 2021). Functional gene annotations were retrieved from the Grapevine Reference Gene Catalogue at https://integrape.eu/resources/genes-genomes/reference-gene-catalogue-and-nomenclature-recommendations/ (last accessed on 18 December 2021).

## Supplementary Materials

The following are available online at https://www.mdpi.com/article/10.3390/biom12020182/s1, Figure S1: qRT-PCR analysis of *DMR6*, and *DLO* genes in grapevine after inoculation with *P. viticola*, Figure S2: Gene network, Table S1: Grapevine *DMR6* and *DLO* gene identifiers in the V1, V2, and V3 annotations, Table S2: Oligonucleotides used in this work, Table S3: Matrix of expression data collected from VESPUCCI relative to different pathosystems, Table S4: Gene network analysis.

## Abbreviations

The following abbreviations are used in this manuscript:

DM	Downy mildew
BTH	Benzothiadiazole
2OG	2-Oxoglutarate
RH	Relative Humidity
NRQ	Normalised Relative Quantity
GA	Gibberellin
