# Supplementary material for: Grapevine DMR6-1 Is a Candidate Gene for Susceptibility to Downy Mildew
Source: Biomolecules. 2022 Jan 22;12(2):182. doi: 10.3390/biom12020182 (PMC8961545; doi:10.3390/biom12020182)
Supplement: Supplementary file 1 [file biomolecules-12-00182-s001.zip › biomolecules-1545379 Supplemental/biomolecules-1545379 figures.pdf]

biomolecules

MDPI

Article

Grapevine DMR6-1 Is a Candidate Gene for Susceptibility to Downy Mildew

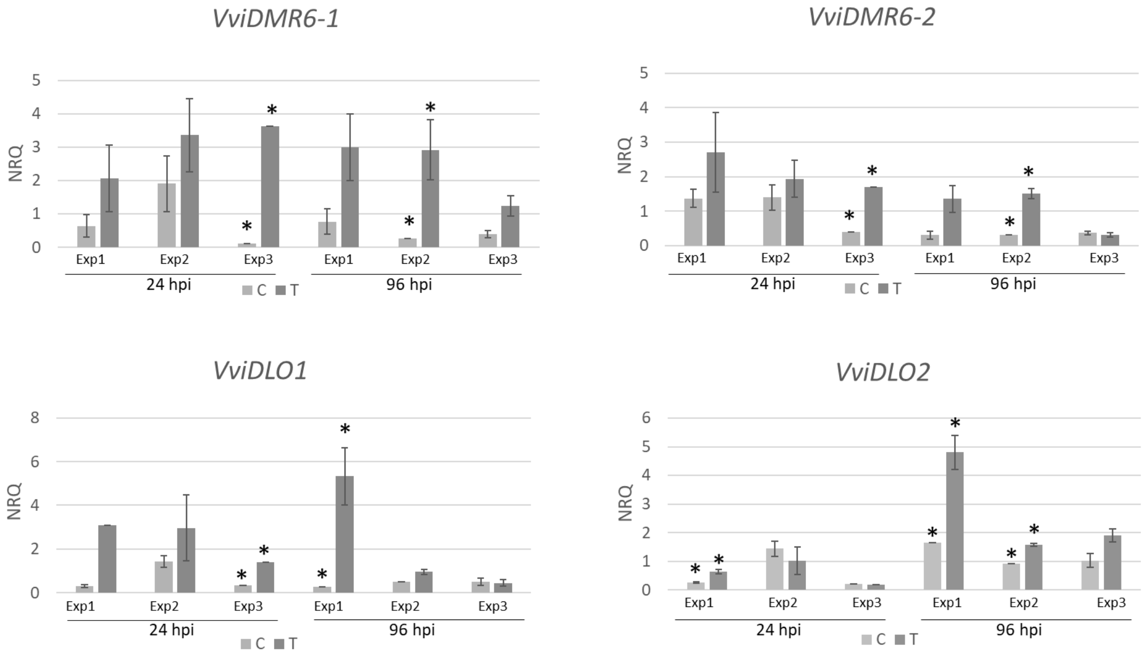

Figure S1. qRT-PCR analysis of DMR6, and DLO genes in grapevine after inoculation with P. viticola.

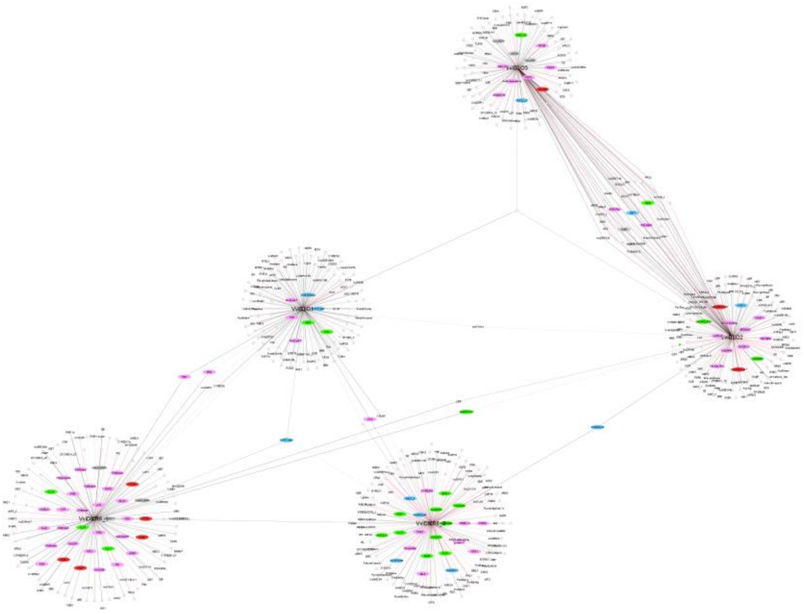

Figure S2. Gene network.
